# Fatal carbon monoxide poisoning in older adults: findings from a 15-year series of forensic autopsies in Turkey

**DOI:** 10.3389/fpubh.2026.1860934

**Published:** 2026-06-26

**Authors:** Samet Kiyak, Yusuf Atan, Gökhan Taşkin, Ramazan Kiyak, Ahmet Sedat Dündar

**Affiliations:** 1 Department of Forensic Medicine, Faculty of Medicine, Balıkesir University, Balikesir, Türkiye; 2 Department of Forensic Medicine, Faculty of Medicine, Bilecik Şeyh Edebali University, Bilecik, Türkiye; 3 Department of Emergency Medicine, Faculty of Medicine, Balıkesir University, Balikesir, Türkiye; 4 Council of Forensic Medicine, Bursa Group Chairmanship, Bursa, Türkiye

**Keywords:** autopsy, carbon monoxide poisoning, carboxyhemoglobin, older adults, forensic toxicology, indoor exposure

## Abstract

**Objective:**

This study aimed to retrospectively evaluate the demographic, toxicological, and pathological characteristics of individuals aged 65 and older who died due to carbon monoxide (CO) poisoning and underwent autopsy.

**Materials and methods:**

Cases aged 65 years and older who were determined to have died from carbon monoxide poisoning at autopsy were analyzed in terms of age, gender, time of death, place of death, source of CO exposure, carboxyhemoglobin levels, toxicological findings, and accompanying chronic diseases.

**Results:**

The mean age of the 115 cases included in the study was 74.6 ± 7.03 years, and 58.3% were male. Sixty percent of deaths occurred during winter months, and 84.3% occurred in domestic settings. The most common source of CO exposure was stoves, accounting for 71.3%. Carboxyhemoglobin levels were mostly in the range of 40–79%, while only 4.3% of cases had carboxyhemoglobin levels above 80%. Various pharmaceutical agents were detected in 43.5% of cases, and ethyl alcohol alone was detected in 4.3% of cases. Among accompanying chronic diseases, cardiovascular system diseases (69.6%) were the most common.

**Conclusion:**

Carbon monoxide poisoning remains an important cause of death among older adults. Fatal cases were predominantly associated with domestic exposure, stove use, and winter months. Cardiovascular and pulmonary comorbidities were common among the victims, highlighting the vulnerability of older populations to fatal carbon monoxide exposure.

## 1 Introduction

Carbon monoxide (CO) poisoning is one of the leading causes of toxicological deaths in Turkey as it is worldwide (1). CO is a colorless, odorless, and tasteless gas that forms as a result of incomplete combustion of organic materials and is often referred to as the “silent killer” due to these characteristics. CO is rapidly absorbed through the respiratory system and binds to hemoglobin with approximately 200–250 times higher affinity than oxygen, forming carboxyhemoglobin (COHb) and thereby preventing oxygen from being transported to tissues (2). This hypoxic process can cause severe damage, particularly in organs with high oxygen consumption such as the brain and heart. Additionally, higher CO-related mortality rates have been observed in older adults (3). The clinical spectrum of CO poisoning can range from mild headaches to loss of consciousness, coma, and death. Older adults are at higher risk of mortality from CO exposure due to reduced physiological reserves, the prevalence of chronic cardiopulmonary diseases, and increased sensitivity to toxic agents (4).

In Turkey, CO poisoning primarily occurs during the winter months and is often the result of improper use of heating devices such as stoves, water heaters, and boilers (4). The province of Bursa is one of the high-risk regions due to its geographical structure and the southerly winds that prevail during the winter months (1). CO exposure in older adults is largely accidental, while CO poisoning for suicidal purposes is quite rare (5). The aim of this study was to retrospectively examine carbon monoxide poisoning–related deaths in individuals aged 65 years and older who underwent autopsy over a 15-year period and to evaluate their demographic characteristics, circumstances of exposure, toxicological findings, and autopsy findings.

## 2 Methods

### 2.1 Study design and participants

This retrospective study included forensic cases aged 65 years and older whose autopsies were performed at the Bursa Group Presidency Morgue Specialisation Department of the Forensic Medicine Institution between January 1, 2010 and December 31, 2024 and whose cause of death was reported as carbon monoxide (CO) poisoning. Permission for the study was obtained from the Education and Scientific Research Commission of the Council of Forensic Medicine, Ministry of Justice, Türkiye (Approval No: 21589509/2025/499, Date: 08/07/2025) and from the Balıkesir University Faculty of Medicine Clinical Research Ethics Committee (Decision No: 2025/9-6, Date: 04/11/2025).

#### 2.1.1 Inclusion criteria

The study included cases aged 65 and older who underwent medico-legal autopsy, where the cause of death was confirmed toxicologically as CO poisoning.

#### 2.1.2 Exclusion criteria

Cases where COHb levels could not be measured, cases where toxicological analysis could not be performed due to advanced postmortem decomposition, and cases where the cause of death was reported as other than CO poisoning were excluded from the study. Additionally, ethanol considered to have resulted from postmortem putrefaction was not interpreted as evidence of ante-mortem alcohol consumption. In accordance with routine forensic toxicology practice, ethanol findings were evaluated together with autopsy findings, decomposition status, and toxicological reports to distinguish postmortem ethanol formation from true ante-mortem alcohol exposure.

#### 2.1.3 Toxicology and COHb analysis

COHb levels were measured using spectrophotometric methods. Toxicological analyses were conducted using gas chromatography–mass spectrometry (GC–MS). All analyses utilized standard reference materials compliant with international validation criteria, and quality control samples were used for verification in each analysis series.

In routine forensic practice, blood samples for COHb analysis are preferentially collected from peripheral vessels, most commonly the femoral vein, during medico-legal autopsy. Samples are transported to the toxicology laboratory under cold-chain conditions and stored at +4 °C until analysis. Following analysis, specimens are retained according to institutional quality assurance and accreditation procedures. In hospitalized cases, available clinical toxicology results and treatment records were also reviewed as part of the medico-legal evaluation. The diagnosis of fatal carbon monoxide poisoning was established through comprehensive assessment of scene investigation findings, autopsy findings, toxicological analyses, and medical records, rather than on the basis of a single COHb threshold value alone.

### 2.2 Data collection

Data related to the cases were obtained by retrospectively reviewing medical records, autopsy reports, incident site investigation reports, toxicological analysis results, and laboratory records. For each case, variables such as age, gender, time of death (year, season, month, day), place of death (home, hospital, outdoor, etc.), source of CO exposure (stove, water heater, fire, etc.), cause of death (CO intoxication alone, CO intoxication + burns), blood COHb level (%), and other toxic substances detected were examined. The cases were classified based on accompanying chronic diseases, autopsy findings, and the available medical history.

Cardiovascular and pulmonary comorbidities were classified based on the combined evaluation of available medical history, medico-legal autopsy findings, and histopathological examination reports. Classification was not based on a single clinical diagnosis but on the overall assessment of documented clinical information together with macroscopic and microscopic pathological findings. Cardiovascular comorbidity classification included chronic cardiovascular conditions documented in medical records and/or supported by autopsy or histopathological findings such as significant coronary atherosclerosis, myocardial hypertrophy, previous myocardial infarction, cardiac dilatation, valvular pathology, or other chronic cardiovascular abnormalities. Pulmonary comorbidity classification included documented chronic respiratory diseases and/or autopsy and histopathological findings consistent with chronic pulmonary pathology, such as pleural thickening, anthracotic changes, pleural adhesions, emphysematous changes, fibrotic alterations, or other chronic pulmonary abnormalities.

The source of CO exposure and the place where death was recorded were evaluated as separate variables. In hospitalized cases, toxicological analyses were performed using blood samples obtained during the clinical course whenever available, in accordance with routine forensic procedures.

For clarity, exposure source, injury mechanism, and certified cause of death were evaluated as separate variables. Fire-related exposure referred to the environmental source of carbon monoxide generation, whereas “CO poisoning with burns” referred to cases in which both carbon monoxide intoxication and thermal injury contributed to death. The certified cause of death was determined according to the final medico-legal autopsy report.

As this was a retrospective study, some variables were not available for all cases. When information regarding a specific variable could not be reliably obtained from forensic records, autopsy reports, toxicological analyses, or medical documentation, the variable was recorded as unavailable and excluded from the relevant descriptive analysis. No imputation methods were applied.

### 2.3 Statistical analysis

The data were compiled using Microsoft Excel 2021 (Microsoft Corp., Redmond, WA, USA) and analyzed using IBM SPSS Statistics for Windows, Version 25.0 (IBM Corp., Armonk, NY, USA). Descriptive statistics for numerical variables are reported as mean ± standard deviation, while categorical variables are reported as number (*n*) and percentage (%).

### 2.4 Ethical principles

This study was conducted in accordance with the ethical principles outlined in the Helsinki Declaration. The identities of the participants in the study were completely anonymized, no personal data was shared, and data confidentiality was maintained.

## 3 Results

A total of 115 cases aged 65 years and older, who underwent autopsy and were determined to have died from carbon monoxide poisoning, were included in the study. The average age of the cases was 74.6 ± 7.03 (min: 65, max: 96) years. A total of 58.3% (*n* = 67) of the cases were male, and 41.7% (*n* = 48) were female.

**Figure 1:**
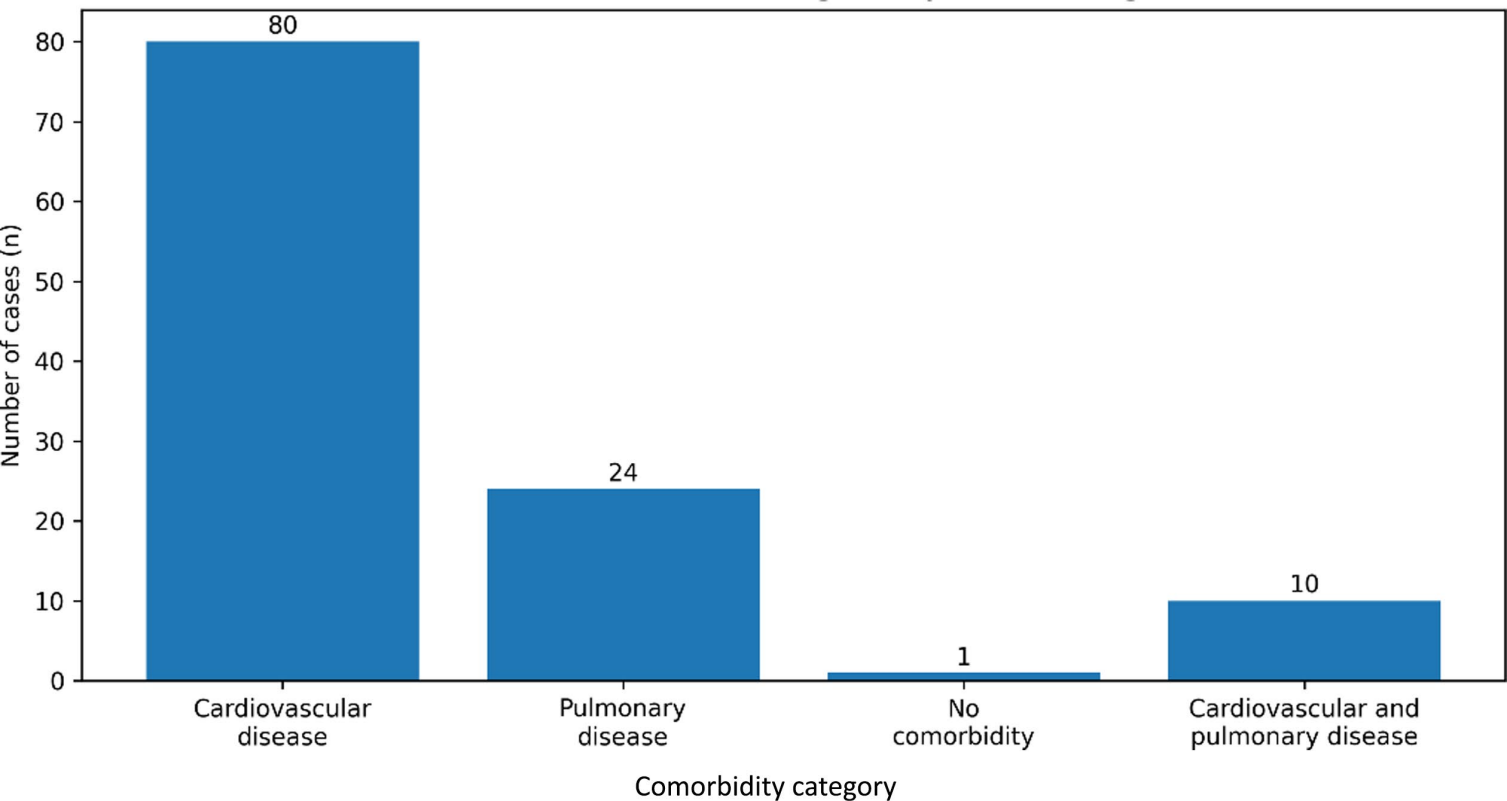
Distribution of comorbid conditions among older adult CO poisoning deaths (*n* = 115). Categories are mutually exclusive. Percentages were calculated using the total study population as the denominator (*n* = 115).

Comorbid conditions were categorized as mutually exclusive groups. Cardiovascular disease alone was present in 80 cases (69.6%), pulmonary disease alone in 24 cases (20.9%), combined cardiovascular and pulmonary disease in 10 cases (8.7%), and no documented comorbidity in 1 case (0.9%) (Figure 1).

**Figure 2:**
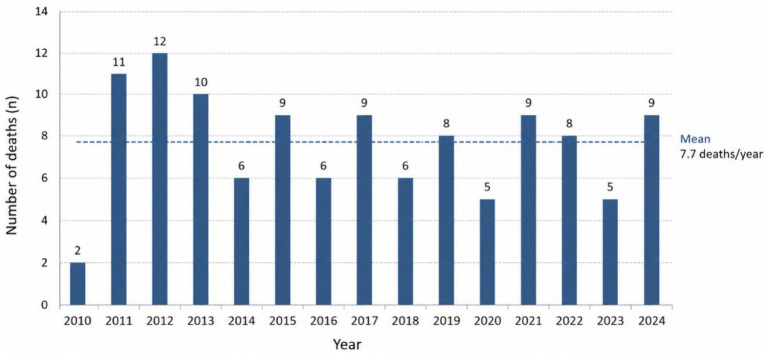
Distribution of carbon monoxide poisoning-related deaths by year.

The annual distribution of carbon monoxide poisoning–related deaths during the study period is presented in Figure 2.

**Figure 3:**
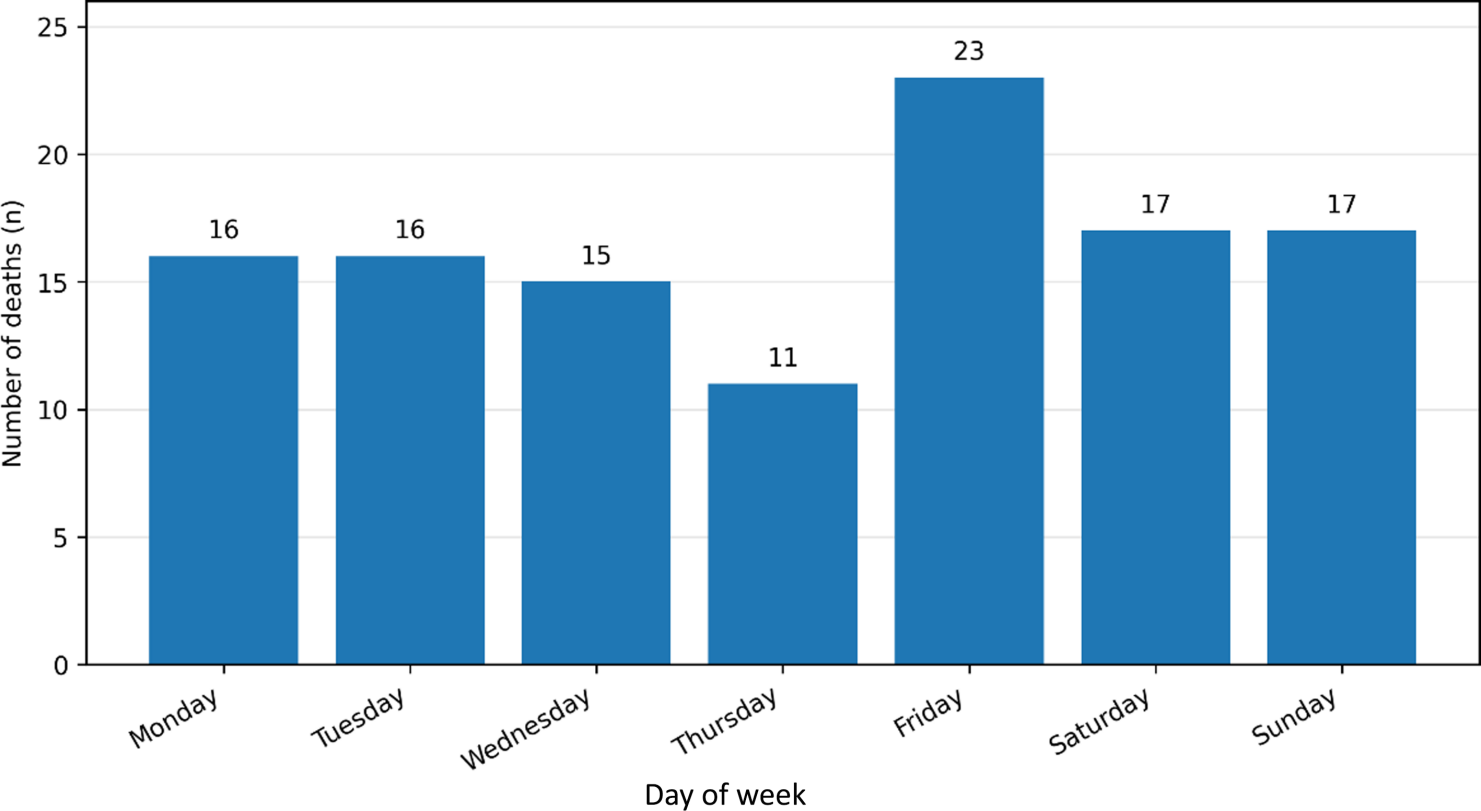
Distribution of carbon monoxide poisoning-related deaths among older adults by day.

The distribution of deaths according to day of the week is presented in Figure 3. Deaths occurred most frequently on Friday, Saturday, and Sunday.

**Figure 4:**
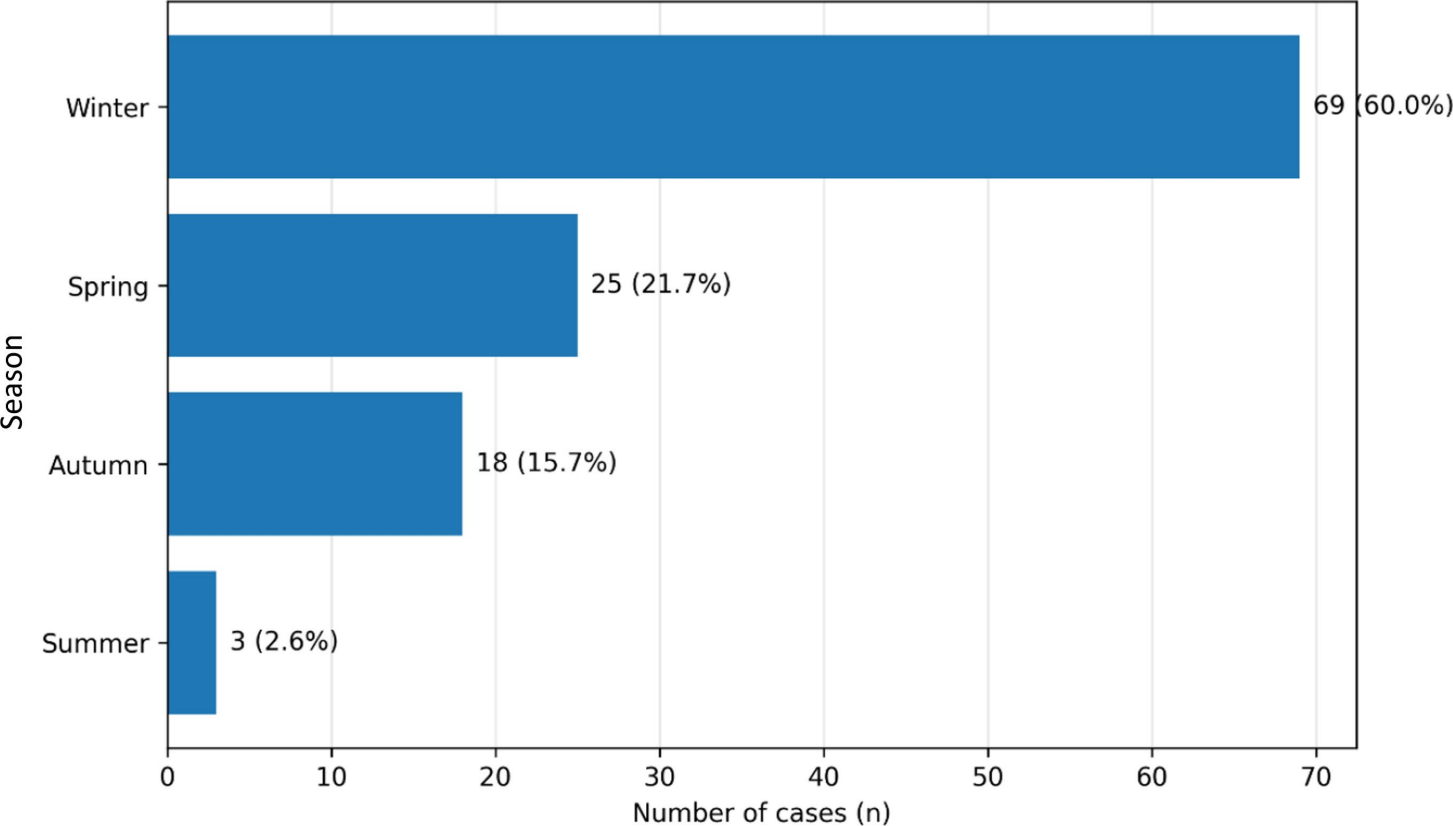
Seasonal distribution of carbon monoxide poisoning-related deaths among older adults.

The seasonal distribution of deaths is presented in Figure 4. Deaths occurred most frequently during the winter months.

The most common source of carbon monoxide poisoning was stoves (71.3%, *n* = 82), followed by fire-related exposure (17.4%, *n* = 20) and water heaters (3.5%, *n* = 4). In nine cases (7.8%), the source of CO exposure could not be identified. Analysis of blood samples taken during medico-legal autopsies revealed that COHb levels were most commonly within the ranges of 40–59% (42.6%, *n* = 49) and 60–79% (39.1%, *n* = 45). Fifteen cases (13.0%) had COHb levels between 20 and 39%, one case (0.9%) had a COHb level between 0 and 19%, and five cases (4.3%) had COHb levels of 80% or higher (Table 1).

**Table 1:** Cause of death and COHb level distribution.

COHb level (%)	Cause of death	
	CO-related death (*n*)	Death related to CO and burns (*n*)
0–19	1^a^ (0.9)	0
20–39	14 (12.2)	1 (0.9)
40–59	44 (38.3)	5 (4.3)
60–79	39 (33.9)	6 (5.2)
≥80	3 (2.6)	2 (1.7)

a One case in the 0–19% category had a documented COHb value of 6.9% obtained during clinical management prior to death. The cause of death was determined through comprehensive forensic evaluation, including scene findings, clinical records, autopsy findings, and toxicological assessment.

**Figure 5:**
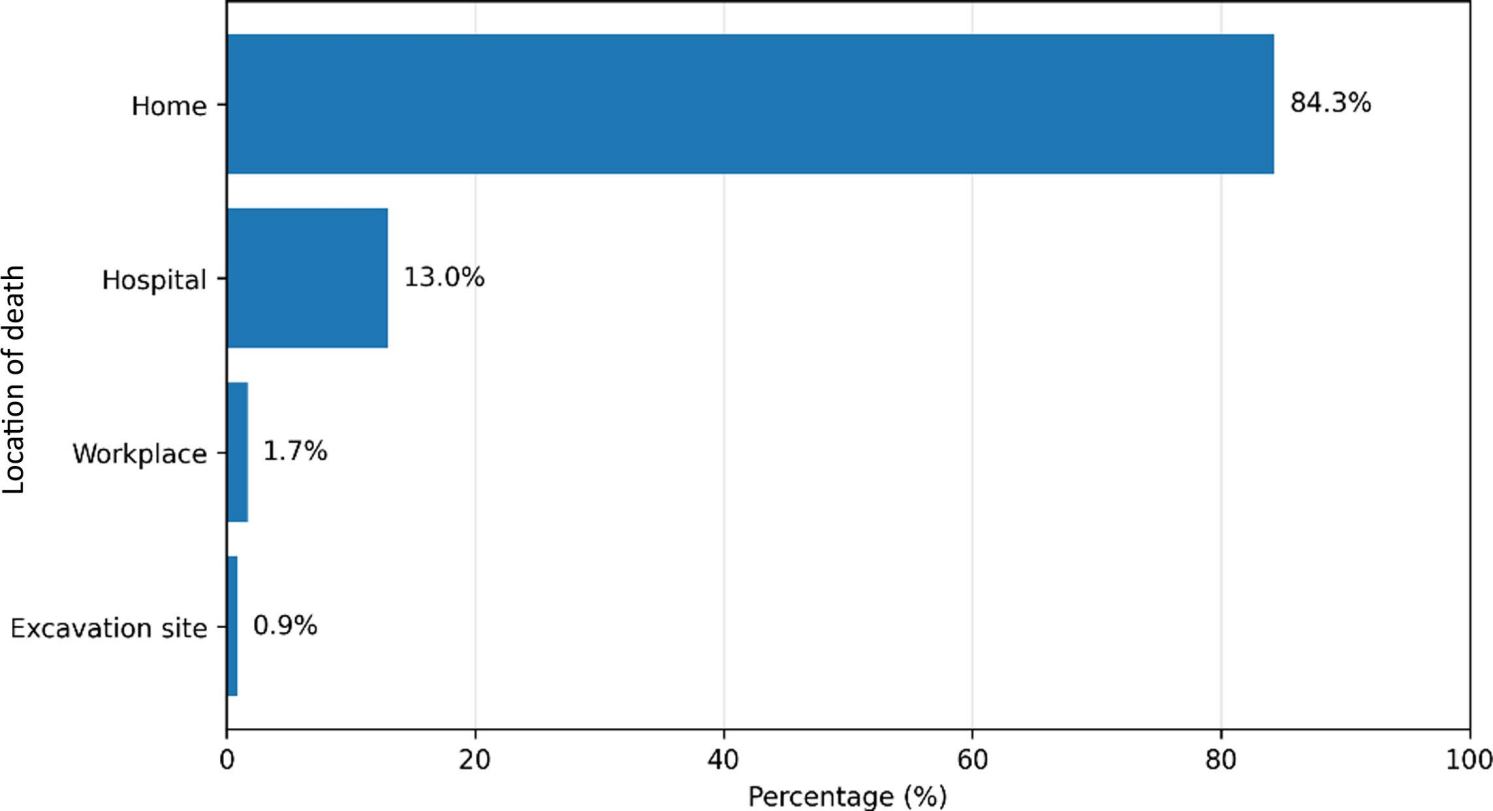
Distribution of the location where death occurred.

Death occurred at home in 84.3% (*n* = 97) of cases. The distribution of locations where death occurred is presented in Figure 5.

In the toxicological analysis results of samples taken during autopsy, active ingredients of medications used to treat various diseases were detected in 43.5% (*n* = 50) of the cases. The number of cases where only ethyl alcohol positivity was detected was 5 (4.3%), while in 60 cases (52.2%), no drugs, alcohol, or narcotic/stimulant substances were detected. The cause of death was carbon monoxide poisoning alone in 86.9% of cases (*n* = 100), while in 13.1% of cases (*n* = 15), death occurred as a result of the combined effect of carbon monoxide poisoning and burns (Table 2).

**Table 2:** Toxicological examination results and distribution of causes of death.

Toxicological result	Cause of death	
	CO-related death	Death related to CO and burns
Pharmaceutical ingredient	42	8
Ethanol	5	0
No drugs, alcohol, or substances detected	53	7
Total	100	15

## 4 Discussion

Carbon monoxide poisoning remains one of the leading toxicological causes of accidental death and continues to represent an important public health concern among older adults (5). In the present study, the mean age of the cases was 74.6 ± 7.03 years, and males accounted for 58.3% of all deaths. Previous studies have reported that reduced physiological reserve, chronic disease burden, polypharmacy, and social vulnerability may influence the outcomes of carbon monoxide poisoning in older adults (1, 6, 7). Consistent with reports from the United States and Sweden, which demonstrated higher CO-related mortality among older men, males constituted the majority of fatal cases in our series (7, 8). This finding suggests that older men may represent a particularly vulnerable subgroup among fatal carbon monoxide poisoning cases.

Annual variation in the number of cases was observed during the study period. Similar fluctuations have been reported in previous studies and may be influenced by environmental and climatic factors, including severe winter conditions (9). Analysis by day of the week showed that 72.2% of deaths occurred on weekdays, with a concentration on Fridays, Saturdays, and Sundays. Given the descriptive nature of the study, no specific explanation for this distribution can be established, and the findings should be interpreted with caution.

The predominance of deaths during the winter months (60%) is consistent with previously reported seasonal patterns of carbon monoxide poisoning (6, 9, 10). Increased use of heating devices and adverse winter conditions have been identified as important contributors to this seasonal distribution (6, 9). The present study demonstrates that 84% of cases occurred in the home environment and that 71% resulted from stove-related CO exposure, indicating that CO poisoning in the older population is largely domestic in origin. Similar findings have been reported in national and international studies, which have consistently identified residential settings and heating devices as the predominant sources of exposure (1, 10–12). The high proportion of stove-related cases observed in the present series may be particularly relevant in regions where climatic and meteorological conditions increase the likelihood of domestic carbon monoxide accumulation (1).

In the majority of our cases, carboxyhemoglobin (COHb) levels were in the range of 40–79%, with only 4.3% of cases showing values above 80%. This finding indicates that fatal cases may occur even in the presence of relatively low measured COHb levels in older adults. In a comprehensive analysis by Hampson and Hauff (13), mortality rates were reported to be significantly higher in CO poisoning cases among older adults with COHb levels above 30% compared to young adults. Additionally, in a prospective cohort analysis by Lee et al. (4), it was found that in older adults, the presence of comorbidities was more predictive of clinical outcomes than the absolute value of COHb levels, and cases resulting in death could occur even at low COHb levels. Furthermore, previous studies have reported that although higher COHb levels may be associated with poisoning severity, COHb concentration alone does not consistently predict clinical severity after adjustment for other factors, highlighting the limited prognostic value of this parameter when interpreted in isolation (14). Our findings emphasize the limited value of COHb levels in predicting mortality in older adults and the importance of carefully evaluating every case where exposure is suspected. In forensic practice, fatal carbon monoxide poisoning should be evaluated using toxicological findings together with autopsy findings, scene investigation data, and environmental circumstances rather than relying on a single laboratory parameter (15).

In 13% of our cases, carbon monoxide (CO) exposure was accompanied by a fire incident. In such cases, mortality cannot be explained solely by COHb levels; inhalation of hot smoke, cyanide, and other toxic combustion products, as well as thermal injury, may also contribute to the mechanism of death. Previous experimental, clinical, and epidemiological studies have shown that smoke inhalation, thermal injury, cyanide exposure, and other combustion products may substantially contribute to mortality in fire-related CO poisoning cases (16–18). These findings suggest that COHb levels alone may be insufficient to assess fatal fire-related CO poisoning and that comprehensive toxicological, autopsy, and clinical evaluation remains essential.

Analysis revealed that various pharmaceutical agents were detected in 43.5% of cases, while ethyl alcohol alone was detected in 4.3% of cases. Previous studies have suggested that medications and toxicological findings may influence the clinical presentation and management of CO poisoning (19, 20). In addition, anticholinergic burden has been associated with adverse outcomes in older adults (21). However, due to the descriptive retrospective design of the present study, the clinical relevance of the detected pharmaceutical agents and their potential contribution to fatal outcomes could not be determined. Therefore, these findings should be interpreted as descriptive toxicological observations rather than evidence of causality, clinically relevant polypharmacy, or drug–CO interaction.

Cardiovascular and pulmonary diseases were commonly observed among older adult victims included in this study. Previous studies have reported that underlying cardiopulmonary disorders may influence the clinical course of carbon monoxide poisoning (4, 22). However, because the present study was descriptive and lacked a comparison group, no causal relationship between comorbidities and mortality risk can be established. The high prevalence of cardiovascular and pulmonary diseases observed in this case series likely reflects the substantial burden of chronic disease in the older population and should be interpreted accordingly. A major strength of this study is the long-term and systematic evaluation of carbon monoxide poisoning–related deaths in older adults based on forensic autopsy data, including demographic, toxicological, and pathological findings. However, the study has some limitations. The retrospective design led to the absence or inadequacy of crime scene investigation reports or detailed medical history information in some cases. Additionally, detailed information regarding functional status, mobility limitation, dependency in daily activities, or bedridden condition was not consistently available in the retrospective forensic records. These factors may influence both the circumstances of CO exposure and the vulnerability of older adults to fatal poisoning and should therefore be considered when interpreting the findings.

## 5 Conclusion

This study describes the environmental and epidemiological characteristics of carbon monoxide poisoning–related deaths among older adults between 2010 and 2024. Stove-related exposure was the predominant source of poisoning and occurred mainly during the winter months and in domestic settings. Cardiovascular and pulmonary diseases were commonly observed among the victims. In addition, several fatal cases were documented with relatively low measured COHb concentrations, particularly among hospitalized individuals who survived long enough to receive medical treatment.

Public awareness: Educational campaigns on carbon monoxide poisoning may be particularly beneficial before the winter season, especially for older adults (23).

Carbon monoxide detectors: Wider use of carbon monoxide detectors in residential settings, particularly in homes occupied by older adults, should be encouraged. Existing Turkish fire safety regulations already include provisions related to gas detection systems, which may contribute to the early recognition of potential CO exposure (24).

High-risk areas: Preventive information regarding the safe use of stoves and chimneys may be particularly relevant in regions where geographical and meteorological conditions increase the risk of carbon monoxide exposure.

Routine consideration of CO exposure in forensic investigations: In older adult decedents, carbon monoxide exposure should be considered when the circumstances of death are compatible with possible poisoning. In addition, low COHb levels do not necessarily exclude CO-related death, particularly in cases with prolonged survival or medical intervention prior to death (22).

## Data Availability

The datasets generated and/or analyzed during the current study are not publicly available because they contain official forensic case records obtained with institutional permission from the Council of Forensic Medicine, Ministry of Justice of the Republic of Türkiye. Access to these data may be considered only upon official application and with approval from the relevant institutional authorities.
